# Strategies for Improving the Stability of Tin‐Based Perovskite (ASnX_3_) Solar Cells

**DOI:** 10.1002/advs.201903540

**Published:** 2020-02-20

**Authors:** Huanhuan Yao, Faguang Zhou, Zhizai Li, Zhipeng Ci, Liming Ding, Zhiwen Jin

**Affiliations:** ^1^ School of Physical Science and Technology & Key Laboratory for Magnetism and Magnetic Materials of MoE & Key Laboratory of Special Function Materials and Structure Design MoE Lanzhou University Lanzhou 730000 China; ^2^ Center for Excellence in Nanoscience (CAS) Key Laboratory of Nanosystem and Hierarchical Fabrication (CAS) National Center for Nanoscience and Technology Beijing 100190 China

**Keywords:** instability, oxidation, perovskite solar cells (PSCs), photovoltaic, tin, tin‐based perovskites, toxicity

## Abstract

Although lead‐based perovskite solar cells (PSCs) are highly efficient, the toxicity of lead (Pb) limits its large‐scale commercialization. As such, there is an urgent need to find alternatives. Many studies have examined tin‐based PSCs. However, pure tin‐based perovskites are easily oxidized in the air or just in glovebox with an ultrasmall amount of oxygen. Such a characteristic makes their performance and stability less ideal compared with those of lead‐based perovskites. Herein, how to address the instability of tin‐based perovskites is introduced in detail. First, the crystalline structure, optical properties, and sources of instability of tin‐based perovskites are summarized. Next, the preparation methods of tin‐based perovskite are discussed. Then, various measures for solving the instability problem are explained using four strategies: additive engineering, deoxidizer, partial substitution, and reduced dimensions. Finally, the challenges and prospects are laid out to help researchers develop highly efficient and stable tin‐based perovskites in the future.

## Graphical abstract

Nontoxic tin‐based perovskite solar cells (PSCs) have attracted attention, but are easily oxidized, which causes their performance and stability to be far behind lead‐based PSCs. Here, strategies to improve the stability of tin‐based PSCs (additive engineering, deoxidizer, partial substitution, and reduced dimensions) are reviewed. Outlooks are also proposed to avoid the shortcoming for fabricating highly efficient and stable PSCs.

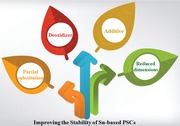

## 1 Introduction

In recent years, perovskite solar cells (PSCs) have developed vigorously, and their power conversion efficiency (PCE) has increased from 3.8% in 2009 to 25.2% in 2019.^[^ 1, 2, 3, 4 ^]^ All currently reported high performance PSCs are based primarily on lead‐based perovskites.^[^ 5, 6, 7, 8 ^]^ However, the toxicity of lead‐based PSCs has received increasing attention, as they may cause harm to the human body and the natural environment.^[^ 9, 10, 11, 12 ^]^ Therefore, developing other perovskite substitutes that reduce lead content is an urgent matter to be able to create environmentally friendly solar cells.

Theoretically, lead can be replaced by metals, such as tin (Sn),^[^ 13, 14 ^]^ bismuth (Bi),^[^ 15, 16 ^]^ and copper (Cu),^[^ 17 ^]^ metalloids germanium (Ge),^[^ 18 ^]^ or antimony (Sb).^[^ 19, 20 ^]^ The most likely alternative is Sn, which is in the same group of elements in the periodic table as lead (Pb).Moreover, the radius of Sn^2+^ (1.35 Å) is smaller than that of Pb^2+^ (1.49 Å), indicating that Sn can replace Pb and retain the original perovskite structure.^[^ 21 ^]^ In addition, compared with the toxicity of lead, Sn^2+^ is oxidized to nontoxic SnO_2_ and SnI_4_ in the tin‐based perovskite material.^[^ 22 ^]^ It also has a similar ns^2^np^2^ electronic configuration as Pb, indicating that the tin‐based perovskite has substantially the same properties as the lead‐based perovskite.^[^ 23 ^]^ To a certain degree, tin‐based perovskites are even better than the lead‐based ones in some optoelectronic properties. For example, the former have a narrower bandgap (*Eg*).^[^ 24 ^]^ The existing high‐efficiency lead‐based PSCs have a wider *Eg* (1.5–1.7 eV),^[^ 25, 26 ^]^ slightly larger than the ideal *Eg* (1.4 eV). By contrast, tin‐based perovskites, such as formamidinium (FA^+^) tin triiodide perovskite (FASnI_3_) (1.41 eV)^[^ 27 ^]^ and CsSnI_3_ (1.3 eV),^[^ 28 ^]^ have a narrower *Eg*, which is more appropriate for achieving efficient PSCs. Tin‐based perovskites also have high electron and hole mobility.^[^ 29 ^]^ For example, the CH_3_NH_3_SnI_3_ compound has electron and hole mobility exceeding 2000 and 300 cm^2^ (V s)^−1^, respectively. High‐optical‐absorption coefficients are another advantage of tin‐based perovskite.^[^ 30 ^]^ However, Sn^2+^ in tin‐based perovskites is unstable and easily oxidized, which deteriorates the semiconductor properties and morphology of the perovskite film, and reduces the efficiency and stability of these materials.^[^ 31, 32, 33 ^]^

This paper summarizes the ways to suppress the oxidation of Sn^2+^ and improve the stability of tin‐based PSCs. First, we discuss the structure and electronic properties of the tin‐based perovskite and our analysis of the reasons for its instability. Next, we explain the recent methods for preparing tin‐based PSCs. The third part details the strategies for solving the instability of tin‐based perovskite, including additive engineering, reducing agent assist, partial substitution, and reducing dimensions. In the last part, we offer our own views and prospects for the future development of tin‐based PSCs.

## 2 Structures and Properties of Tin‐Based Perovskite

### 2.1 Crystalline Structure

**Figure 1:**
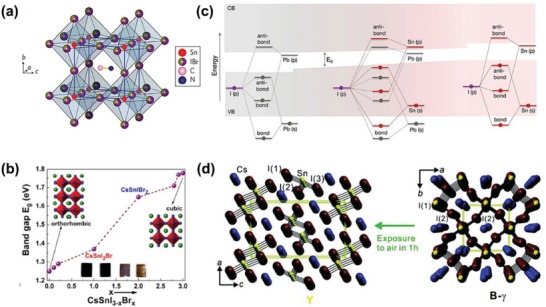
a) Crystal structure of CH_3_NH_3_SnI_3−_ *_x_*Br*_x_* perovskite. Reproduced with permission.^[^ 34 ^]^ Copyright 2014, Nature Publishing Group. b) Bandgap variation with Br^−^ concentration. Reproduce with permission.^[^ 43 ^]^ Copyright 2015, American Chemical Society Publications. c) Schematic representation of the origin of the bandgap in MAPbI_3_, MA(Pb_0.75_Sn_0.25_)I_3_, MA(Pb_0.25_Sn_0.75_)I_3_, MASnI_3_.The shaded area indicates the valence band and the conduction band, and the thick line indicates the molecular orbital map. Reproduce with permission.^[^ 46 ^]^ Copyright 2018, American Chemical Society Publications. d) The crystal structure changes from orthorhombic (B‐γ) to yellow (Y) after exposure to air at 300 K. Reproduce with permission.^[^ 51 ^]^ Copyright 2012, American Chemical Society Publications.

The crystal structure of tin‐based perovskites is similar to that of Pb‐based perovskites ABX_3_, with the A site cations occupying the cubic cavity in the [BX_6_] (B = Sn, Pb) octahedron and the B atoms filling the octahedral void. For example, methylammonium (MA^+^) tin triiodobromide perovskite (MASnI_3−_ *_x_*Br*_x_*) is constructed by a network of SnX_6_ (X= I, Br) octahedra that encompass an organic cation [CH_3_NH_3_]^+^, as shown in **Figure** **1**a.^[^ 34 ^]^

The stability of the perovskite structure can be assessed using the Goldschmidt tolerance factor (*t*) (Equation (1))^[^ 35, 36, 37 ^]^

(1) $$t\;=\frac{r_{\text{A}}+r_{\text{X}}}{\sqrt{2}\left(r_{\text{B}}+r_{\text{X}\;}\right)}$$

Where *r*_A_, *r*_B_, and *r*_X_ are the ionic radii of the A, B, and X positions, respectively. When *t* is between 0.813 and 1.107, the perovskite structure is in a stable state, and cubic perovskite is formed when the *t* value is between 0.9 and 1.0.^[^ 38, 39 ^]^ Therefore, adjusting *t* can structurally optimize the stability in tin‐based perovskite.

Changing the A ion can be used to adjust the crystal structure. For example, under ambient conditions, MASnI_3_ is a pseudocubic tetragonal (P4mm) crystal structure,^[^ 40 ^]^ whereas FASnI_3_, a orthorhombic structure.^[^ 41 ^]^ At room temperature (RT), CsSnI_3_ shows a black phase (B‐γ) 3D orthogonal crystal structure. When exposed to air, CsSnI_3_ changes to the Y phase.^[^ 42 ^]^ Its crystal structure can also be adjusted by changing the X^−^ ions. By incorporating Br anion into CsSnI_3−_ *_x_*Br*_x_*, the crystal structure transforms from orthorhombic (CsSnI_3_) to cubic (CsSnBr_3_), as shown in Figure 1b.^[^ 43 ^]^

### 2.2 Electronic Structure

The theoretical calculations of the electronic structure are useful for analyzing the optoelectronic properties of the device. By using the mixed density functional theory calculation, we can calculate the electronic structure of the tin‐based perovskite. Studies have been found that the electronic structures of ASnX_3_ have common features.^[^ 44, 45 ^]^ In theory, for MASnI_3_, the valence band maximum (VBM) is composed mainly of antibonding hybridized Sn s and I p orbitals, with dominant contributions from I p, whereas the conduction band minimum (CBM) is determined by antibonding mixing of Sn p and I p orbitals, with major contribution from Sn p, as shown in Figure 1c.^[^ 46 ^]^ By studying CH_3_NH_3_SnBr_3_ further, Bernal and Yang found that the *Eg* is mainly determined by the bond of Sn—Br, whereas the organic A cation does not participate in the formation of the VB and CB, and its function is to provide electrons in the perovskite material.^[^ 44 ^]^ In α‐CsSnI_3_ (cubic phase), Huang and Lambrecht also obtained similar results.^[^ 45 ^]^ Feng and Xiao confirmed these results by changing the *Eg* from 1.67 to 3.0 eV in the MASnX_3_ perovskites by changing X.^[^ 47 ^]^ In addition, as the binding strength of the Sn‐s and Sn‐p atomic orbitals is less than the corresponding Pb states, as shown in Figure 1c, the energy band edges of pure MASnI_3_ are bound less strongly than those of pure MAPbI_3_, which also leads to the reduction of the *Eg* of tin‐based perovskite. For example, The FASnI_3_ possesses a smaller *Eg* (1.41 eV)^[^ 27 ^]^ than FAPbI_3_ (1.48 eV)^[^ 48 ^]^ or MAPbI_3_ (1.5 eV).^[^ 25 ^]^ CsSnI_3_ also has a relatively small *Eg* (1.3 eV) ^[^ 28 ^]^ than CsPbI_3_ (1.7 eV).^[^ 26 ^]^

### 2.3 Performance and Instability

The highest PCE reported for a pure tin‐based PSC based on FASnI_3_ is 9.6%, which is still much lower than that of lead‐based PSCs.^[^ 49 ^]^ The root cause is that tin‐based PSCs have many disadvantages compared with high‐performance lead‐based PSCs. First, when exposed to air or only in glovebox with ultrasmall amount of oxygen, Sn^2+^ is easily oxidize to Sn^4+^.^[^ 50 ^]^ For example, when at near room temperature, Sn^2+^ is easily oxidized and CsSnI_3_ is prone to phase change, thereby producing Cs_2_Sn_2_I_6_ with high density of Sn^4+^ defects, as shown in Figure 1d.^[^ 51 ^]^ Second, as Sn^2+^ is easily oxidized to Sn^4+^, it will make Sn^2+^ easily lost during film formation, and high‐density Sn vacancies act as p‐type metal with high carrier density; this process causes severe recombination, thereby reducing device performance and repeatability.^[^ 52 ^]^ Third, in addition to the chemical instability mentioned above, there are problems with tin‐based perovskite films. Owing to the greater Lewis acidity of Sn^2+^ versus Pb^2+^, SnI_2_ reacts faster with CH_3_NH_3_I_3_ to form perovskites, which hinders uniform growth of the film.^[^ 40 ^]^ These shortcomings lead to instability of the tin‐based PSCs, which, in turn, reduces their efficiency. In the fourth part of this paper, we discuss in detail the recent strategies to solve the instability of tin‐based perovskites.

## 3 Fabrication Methods

Uniform and dense films are critical for tin‐based PSCs. The most common preparation methods include all solution, evaporation, and evaporation assist solution methods. In Subsections 3.1–3.3, we discuss the ways to obtain excellent film quality in the preparation methods.

### 3.1 All Solution Method

In general, the solution method is advantageous because it can prepare and operate easily and is relatively low‐cost. The disadvantage is that it is difficult to prepare in a large‐area, high‐efficiency equipment because of uncontrollable factors, such as airflow speed and antisolvent dripping. Generally, the spin‐coating processing can be divided into one‐step and two‐step methods.^[^ 53 ^]^

**Figure 2:**
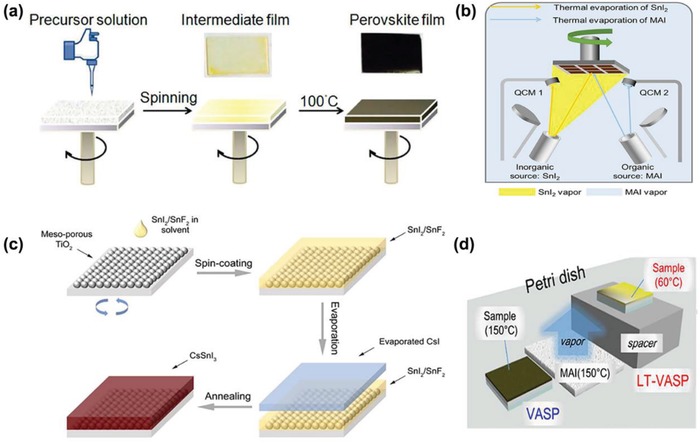
a) A one‐step spin coating process forms a DMSO solvate intermediate. An image of the film before and after the annealing process. Reproduce with permission.^[^ 40 ^]^ Copyright 2015, American Chemical Society Publications. b) Schematic diagram of the process of hybrid thermal evaporation to form MASnI_3_. Reproduced with permission.^[^ 58 ^]^ Copyright 2016, The Royal Society of Chemistry. c) Schematic of the evaporation‐assisted solution method. Reproduced with permission.^[^ 61 ^]^ Copyright 2018, Wiley‐VCH Publications. d) Schematic of LT‐VASP method. Reproduce with permission.^[^ 63 ^]^ Copyright 2016, American Chemical Society Publications.

During the one‐step method, the sample of the ASnX_3_ precursor is dissolved in a specific solvent, such as N,N‐dimethylformamide and dimethyl sulfoxide (DMSO) in a certain ratio and the precursor solution is spin‐coated by centrifugal force, as shown in **Figure** **2**a.^[^ 40 ^]^ During the spin‐coating process, a common method involves adding an antisolvent; its dripping is a key process in producing a high quality perovskite film. Common antisolvents include diethyl ether (DE), toluene (TL), and chlorobenzene (CB).^[^ 54 ^]^ In 2016, Ke et al. used DE as an antisolvent to prepare a uniform and pinhole‐free perovskite film.^[^ 55 ^]^ Later, Fujihara et al. used a mixture of TL and hexane as an antisolvent to explore a flat perovskite layer that can achieve high‐surface coverage by varying the extraction rate.^[^ 56 ^]^

Owing to the rapid reaction of SnI_2_ to CH_3_NH_3_I_3_, the two‐step method for tin‐based perovskites is rarely used.^[^ 40 ^]^ In 2018, Zhu et al. introduced the two‐step method, in which the SnY_2_‐TMA complex (Y = I^−^, F^−^) was formed in the first step by spin coating, and then reacted to FAI by ion exchange to convert it into FASnI_3_.^[^ 23 ^]^

### 3.2 All Vapor Deposition

Typically, vapor deposited perovskite films are more uniform and have higher surface coverage than solution treated films.^[^ 57 ^]^ For tin‐based perovskites, Yu et al. used coevaporation to prepare a MASnI_3_ film with excellent surface coverage and compact morphology, as shown in Figure 2b.^[^ 58 ^]^ As a result, the best PCE is 1.7% and the high open circuit voltage (*V*_OC_) is 377 mV. In the same year, Jung et al. deposited CH_3_NH_3_SnBr_3_ perovskite films by coevaporation and sequential evaporation of SnBr_2_ and CH_3_NH_3_Br.^[^ 59 ^]^ In the sequential evaporation, the maximum efficiency was obtained at 1.12% due to the protective effect of the top MABr layer, which prevented oxidation in the air. Recently, Ball et al. prepared a low *Eg* FA_1−_ *_x_*Cs*_x_*Sn_1−_ *_y_*Pb*_y_*I_3_ perovskite film by dual source coevaporation and obtained a uniform perovskite film with an efficiency of 10%.^[^ 60 ^]^

### 3.3 Evaporation Assist Solution Method

In 2018, Zhu et al. prepared a fairly uniform, dense and pinhole‐free CsSnI_3_ film using vapor assisted solution method (VASP).^[^ 61 ^]^ In this method, SnF_2_ and SnI_2_ are, first, deposited on a substrate, and then, after annealing, they are transferred to a vacuum chamber for CsI deposition. Finally, the evaporated sample is annealed at 150 °C to ensure mutual diffusion of components, as shown in Figure 2c. Xi et al. also prepared uniform FASnI_3_ films using the same method.^[^ 62 ^]^ First, they spin‐coated FAI/polymer layers and then evaporated the SnI_2_ layer. In this method, the introduced polymer significantly inhibits the fine FAI crystals and provides various interdiffusion pathways to react completely to SnI_2_. As a result, 3.98% efficiency was obtained without any additives.

Compared with the traditional VASP method, Yokoyama et al. prepared CH_3_NH_3_SnI_3_ films through a low‐temperature VASP (LT‐VASP).^[^ 63 ^]^ In this method, methylammonium iodide (MAI) powder is placed on a high temperature petri dish; a SnI_2_ film is deposited on the substrate by the reaction of MAI (gas)‐SnI_2_ (solid) with the optimal temperature of the solid‐state SnI_2_ substrate being at 60 °C, as shown in Figure 2d. They obtained a uniform and dense film, and the best efficiency was 1.86%. In the following year, they used the same method to prepare MASnI_3−_ *_x_*Br*_x_* films and achieved lower hole doping levels and better air stability.^[^ 64 ^]^

## 4 Strategies for Improving Stability

To increase the stability of tin‐based perovskites, reducing the oxidation of Sn^2+^ and obtaining uniform and smooth films are necessary. To sum up, there are four major strategies to increase the stability of tin‐based perovskites:

1) Additive engineering
2) Deoxidizer
3) Partial substitution
4) Reduced dimensions

### 4.1 Additives Engineering

Additives play a role in optimizing morphology and adjusting photoelectron properties, which can be mainly divided into three main types: inorganic, organic, and intermediate. The photovoltaic parameters of different additives are summarized in **Table** **1**, where PCE means the photoelectric conversion efficiency and sPCE represents the stable PCE. In the following subsections, we describe the role of different additives from different aspects.

**Table 1:** Photovoltaic parameters of ASnX_3_ PSCs fabricated with different additives

Additives		Configuration	*J*_SC_ [mA cm^−2^]	*V*_OC_ [V]	FF [%]	PCE [%]	sPCE [%]	Ref.
Inorganic	SnF_2_	FTO/TiO_2_/CsSnI_3_/Spiro or m‐MTDATA/Au	22.70	0.24	37.00	2.02	—	^[^ 13 ^]^
		FTO/TiO_2_/CsSnBr_3_/Spiro/Au	9.00	0.41	58	2.10		^[^ 66 ^]^
		FTO/TiO_2_/FASnI_3_/Spiro/Au	24.45	0.238	36.00	2.10	1.41	^[^ 27 ^]^
		FTO/TiO_2_/MASnIBr_2_/Spiro/Au	13.78	0.45	57.30	3.70	3.46	^[^ 68 ^]^
		ITO/PEDOT:PSS/FASnI_3_/C_60_/BCP/Ag	22.07	0.465	60.67	6.22	6.00	^[^ 41 ^]^
		ITO/PEDOT:PSS/(FA)_0.75_(MA)_0.25_SnI_3_/C_60_/BCP/Ag	21.20	0.61	62.70	8.12	7.29	^[^ 69 ^]^
		ITO/PEDOT:PSS/(FA)_0.75_(MA)_0.25_SnI_3_/C_60_/BCP/Al	24.30	0.55	67.30	9.06	8.26	^[^ 54 ^]^
	Others	ITO/CsSnI_3_ +SnCl_2_/PC_61_BM/C_60_/Ag	9.89	0.50	68.00	3.56	3.35	^[^ 30 ^]^
		FTO/TiO_2_/CsPbI_3_+SnBr_2_/PTAA/Au	18.50	0.44	52.90	4.33	—	^[^ 70 ^]^
		ITO/CuI/CsSnI_3_+SnI_2_/C_60_/BCP/Al	8.5	0.465	54	2.13		^[^ 71 ^]^
		FTO/TiO_2_/FASnI_3_+SnI_2_/PTAA/Au	25.71	0.381	49.05	4.81		^[^ 72 ^]^
Organic		ITO/PEDOT:PSS/FASnI_3_+EDAI_2_/C_60_/BCP/Ag	20.00	0.516	71.60	7.40	6.40	^[^ 74 ^]^
		ITO/PEDOT:PSS/GA_0.2_FA_0.8_SnI_3_+EDAI_2_/C60/BCP/Ag	20.80	0.562	72.60	8.50	7.40	^[^ 75 ^]^
		ITO/PEDOT:PSS/FASnI_3_+5‐AVAI/PCBM/BCP/Ag	18.89	0.59	62.00	7.00	5.80	^[^ 78 ^]^
		FTO/TiO_2_/{en}FASnI_3_/PTAA/Ag	22.54	0.48	65.96	7.14	6.05	^[^ 82 ^]^
		FTO/TiO_2_/{en}MASnI_3_/PTAA/Au	24.28	0.428	63.72	6.63		^[^ 83 ^]^
		FTO/TiO_2_/{en}CsSnI_3_/PTAA/Au	25.07	0.281	53.82	3.79		^[^ 83 ^]^
		ITO/PEDOT:PSS/FASnI_3_+TFEAC/PC_61_BM/BCP/Al/Ag	22.07	0.40	60.00	5.30	—	^[^ 84 ^]^
Intermediate phase		FTO/TiO_2_/MASnI_3_/Spiro/Au	15.20	0.668	57.00	5.79		^[^ 40 ^]^
		FTO/TiO_2_/FASnI_3_/Spiro/Au	23.70	0.32	63.00	4.80	3.71	^[^ 87 ^]^
		ITO/SnO_2_/C_60_/FASnI_3_+SnF_2_+TMA/Spiro/Ag	21.65	0.31	64.70	4.34	4.02	^[^ 23 ^]^
		ITO/PEDOT:PSS/FASnI_3_+SnF_2_+TMA/C_60_/Ag	22.45	0.47	67.80	7.09	6.79	^[^ 23 ^]^

#### 4.1.1 Inorganic

Inorganic additives are mainly Sn halides and affect the performance of tin‐based perovskites from the following four aspects:

1) Inhibiting the oxidation of Sn^2+^ and reducing Sn‐cation vacancies and background carrier density
2) Optimizing the position of the energy level
3) Improving film morphology and enhancing stability
4) Tuning crystal phases

##### SnF_2_

**Figure 3:**
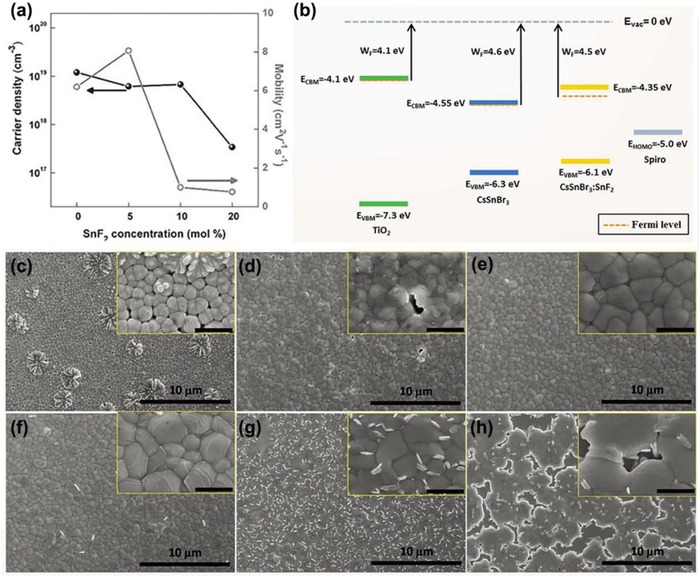
a) Hall measurement of CsSnI_3_ films at various SnF_2_ concentrations. Reproduced with permission.^[^ 13 ^]^ Copyright 2014, Wiley‐VCH Publications. b) Energies of the VBM, CBM, and the Fermi level of dense TiO_2_, pristine CsSnBr_3_, CsSnBr_3_ (with 20 mol% SnF_2_).^[^ 66 ^]^ Copyright 2016, American Chemical Society Publications. SEM images of FASnI_3_ films with different SnF_2_ concentrations: c) 0 mol%, d) 5 mol%, e)10 mol%, f) 15 mol%, g) 20 mol%, h) 30 mol%. Reproduced with permission.^[^ 41 ^]^ Copyright 2016, Wiley‐VCH Publications.

SnF_2_ is commonly used as an antioxidant.^[^ 27 ^]^ Its role may be to reduce the degree of oxidation and create/maintain a reducing environment; it is usually added to tin‐based perovskite preparation solutions.^[^ 65 ^]^ CsSnI_3_ has a high carrier concentration at room temperature of about 10^17^ cm^−3^ and a hole mobility of about 585 cm^2^ V^−1^ s^−1^; these elements indicate p‐type conductivity.^[^ 51 ^]^ The root cause of this phenomenon is the intrinsic defect associated with Sn vacancy. To reduce background carrier density, Kumar et al. added SnF_2_ into CsSnI_3_.^[^ 13 ^]^ As a result, an increase in SnF_2_ causes a decrease in the carrier density of CsSnI_3_, as shown in **Figure** **3**a. This finding indicates that SnF_2_ can reduce the concentration of Sn vacancies, thereby reducing metal conductivity. Later, Koh et al. obtained the same result and found that SnF_2_ delayed the oxidation of Sn^2+^.^[^ 27 ^]^ In addition, Gupta et al. also found that SnF_2_ could optimize band alignment at the perovskite interfaces (as shown in Figure 3b) and improve the stability of X‐ray beam damage, thereby increasing its stability in the experiment.^[^ 66 ^]^ In addition, adding SnF_2_ to CsSnI_3_ can also improve light stability and help form an effective light emitter.^[^ 67 ^]^

For tin‐based PSCs, high quality films are critical to reducing defects, for an excellent film can prevent oxygen and moisture from penetrating into its internal region and then reduce oxidation of Sn^2+^. To improve film morphology, Koh et al. reported that SnF_2_ inhibits the oxidation of Sn^2+^ and improves the morphology and substrate coverage of FASnI_3_ perovskite.^[^ 27 ^]^ In 2016, Liao et al. prepared an inverted planar device architecture. They found that the flower‐like structure and pinholes on the film were disappearing with the SnF_2_ increasing, and when the proportion of SnF_2_ increased from 10% to 20%, the film became denser and more uniform, as shown in Figure 3c–h.^[^ 41 ^]^ Xiao et al. explored its effects by preparing MASnIBr_2_ films with different concentrations of SnF_2_.^[^ 68 ^]^ They found that SnF_2_ will, first, precipitate to produce more crystal growth cores, so that the film can be more uniform and the coverage is higher. Inspired by this idea, in 2017, Zhao et al. fabricated (FA)_0.75_(MA)_0.25_SnI_3_ PSCs with 10 mol% SnF_2_ additive; they achieved an optimal PCE of 8.12%. When stored in a nitrogen glove box for 400 h, it can maintain about 80% of the original PCE.^[^ 69 ^]^ Later, Liu et al. examined the effects of different antisolvents on film formation. They prepared FA_0.75_MA_0.25_SnI_3_ with SnF_2_ as additives and DE, toluene (TL), CB as antisolvent.^[^ 54 ^]^ As result, CB as antisolvent obtained a dense and uniform film.

Many studies have shown that the addition of SnF_2_ also prevents the formation of unwanted phases. For example, the black CsSnI_3_ perovskite phase becomes a yellow nonperovskite phase Cs_2_SnI_6_ in the air due to the oxidation of Sn^2+^. The addition of SnF_2_ prevents the formation of nonperovskite in CsSnI_3._^[^ 13 ^]^ For CsSnBr_3_, the formation of this extra phase CsSn_2_Br_5_ also can be eliminated by adding SnF_2_.^[^ 43 ^]^

##### Others

**Figure 4:**
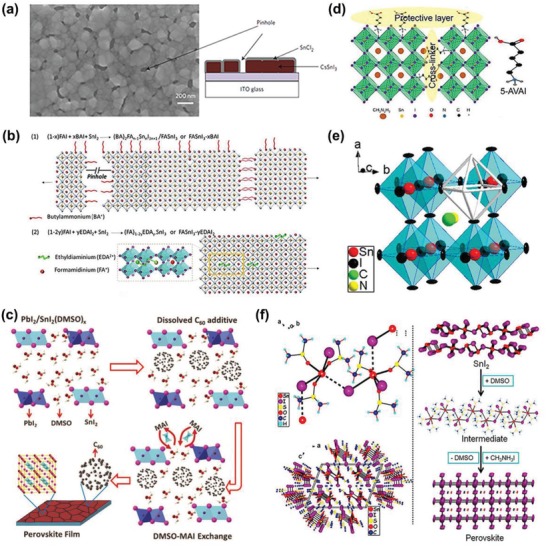
a) The left panel is an SEM image of a CsSnI_3_ film prepared with a 10 mol% SnCl_2_ additive. The picture on the right shows a thin film structure covered with a thin SnCl_2_ layer. Reproduced with permission.^[^ 30 ^]^ Copyright 2016, Nature Publishing Group. b) Schematic representation of perovskites in the presence of BAI and EDAI_2_ additives. Reproduced with permission.^[^ 74 ^]^ Copyright 2018, The Royal Society of Chemistry. c) Schematic diagram of C60 additive assisted crystallization of MAPb_1−_ *_x_*Sn*_x_*I_3_ film. Reproduced with permission.^[^ 77 ^]^ Copyright 2017, The Royal Society of Chemistry. d) Schematic diagram of 5‐AVAI as a protective layer on the PSC of FASnI_3_. Reproduce with permission.^[^ 78 ^]^ Copyright 2019, American Chemical Society Publications. e) Schematic diagram of a hypothetical unit cell of a hollow perovskite with SnI_2_ vacancies. Reproduce with permission.^[^ 83 ^]^ Copyright 2017, American Chemical Society Publications. f) The left panel shows the structure of SnI(DMSO)_3_^+^ ions and the unit cell of SnI_2_•3DMSO by isolated I^−^ ions. The image to the right shows the formation of a perovskite film from SnI_2_ to SnI_2_•3DMSO intermediate. Reproduce with permission.^[^ 40 ^]^ Copyright 2015, American Chemical Society Publications.

In addition to SnF_2_, other Sn halides, such as SnBr_2_, SnCl_2_, and SnI_2_, can also improve the performance of tin‐based PSCs. In 2016, Marshall et al. examined how SnX_2_ (X = F, Cl, Br, I) additives affect the stability of CsSnI_3_ solar cell.^[^ 30 ^]^ Experiments have shown that the solar cell had high efficiency (3.56%) and good stability when added with SnCl_2_. Unpackaged devices have been tested in ambient air at a humidity of about 25%. After 7 h of simulated sunlight under constant 1 sunlight, the PCE is only reduced to 70%. They explained that SnCl_2_ existed as a thin film or layer of particles at the perovskite crystalline, as schematically illustrated in **Figure** **4**a. The SnCl_2_ surface layer acts as a desiccant and sacrificial agent because SnCl_2_ absorbs H_2_O to form a stable hydrate (SnCl_2_·2H_2_O), which is then oxidized to SnO_2_. Heo et al. also proved that SnX_2_ (X = F, Cl, Br) could passivate the surface effectively.^[^ 70 ^]^ However, the difference is that SnBr_2_ is the most effective additive, which can stay stable for 100 h at the maximum PCE (4.3%).

For SnI_2_, in 2015, Marshall et al. used excess SnI_2_ during CsSnI_3_ synthesis and achieved a 2.76% PCE, together with a 0.55 V *V*_OC_, based on a based on a CuI|CsSnI_3_|fullerene p–i–n structure.^[^ 71 ^]^ Inspired by this idea, Song et al. introduced SnI_2_ as an excess Sn^2+^ compound in the ASnI_3_ perovskite and combined it with a reducing atmosphere.^[^ 72 ^]^ As a result, excess SnI_2_ did not affect the formation of the perovskite phase and provided more Sn^2+^ to the system, compensating for the Sn^2+^ lost during its oxidation to Sn^4+^. The optimal PCE of CsSnI_3_ was increased to 4.81%.

Superhalides [BH_4_]^−^ and [AlH_4_]^−^ are inorganic additives with high electron affinity. In 2018, Xiang et al. introduced it into the MASnI_3_ perovskites and proposed a new type of organic metal super halide perovskites, called MASnI_2_BH_4_ and MASnI_2_AlH_4_.^[^ 73 ^]^ As a result, the oxidation resistance of Sn^2+^ in the MASnI_3_ perovskite could be improved because of a large amount of electrons transfer between Sn^2+^ and [BH_4_]^−^/[AlH_4_]^−^.

#### 4.1.2 Organic

Organic additives are often used in precursor solutions. The role of organic cations is as follows:

1) Control the morphology of the film by kinetic equilibrium between nucleation and crystal growth
2) Passivate crystal surface to reduce Sn^2+^ oxidation
3) Reduce defects for better charge separation
4) Form a 3D hollow perovskite structure

BAI (Butylammonium iodide) and EDAI_2_ (ethylenediammonium diiodide) are two organic cationic salts. In 2018, Jokar et al. prepared FASnI_3−_ *_x_*BAI and FASnI_3−_ *_y_*EDAI_2_ by adding BAI and EDAI_2_, respectively.^[^ 74 ^]^ As a result, these two cations (BA^+^ and EDA^2+^) changed the morphology of the film in different ways, reduced the defect state, and enhanced the crystallinity of the perovskite, as shown in Figure 4b. Two organic salts also prevented oxidation of Sn^2+^ to form Sn^4+^. The best PCE was 7.4% for FASnI3‐EDAI2 1% when it was made the first day. However, because of the slow passivation, efficiency slowly increased during the storage process. The best performance was achieved after storing for 1400 h (PCE 8.9%), with only slight degradation if stored beyond 2000 h. In the next year, Jokar et al. incorporated guanidinium (GA^+^) into the FASnI_3_ crystal structure with EDAI_2_ as an additive,^;^ they obtained the same result.^[^ 75 ^]^ The small amount of EDAI_2_ stabilized the perovskite tin and prevented the oxidation of Sn^2+^ on the surface. The device PCE reached 9.6% (after storing for 2000 h) and our cell was stable, even though verification proceeded under strict conditions.

Next, we discuss some organic additives that can form protective layers like SnCl_2_, as mentioned above.^[^ 30 ^]^ For example, C_60_ can not only enhance charge transport properties but also passivate the grain boundaries and surfaces of perovskite layer.^[^ 76 ^]^ In 2017, Liu et al. used C_60_ as additive in the hybrid Sn‐Pb precursor solution.^[^ 77 ^]^ As a result, C_60_ was distributed throughout the grain boundary of perovskite layer and used as a barrier to resist moisture and oxygen in the air effectively, as shown in Figure 4c. After 7 d of exposure to air, the PCE of unpackaged solar cells was only attenuated by 20%. Later, Kayesh et al. found that 5‐pentyl ammonium iodide (5‐AVAI) can also act as a protective layer.^[^ 78 ^]^ It is a hydrophobic long carbon chain organic additive with a bifunctional group, which can improve the stability of lead‐based perovskite by crosslinking adjacent grains to form a protective layer.^[^ 79 ^]^ They prepared FASnI_3_ by using 5‐AVAI as additive. As a result, they found that 5‐AVAI synergized with SnI_6_^4−^ through hydrogen bond formation, which was used as a “line” for sewing grain boundaries and formed an inert protective layers, as shown in Figure 4d. The PCE also improved from 3.4% to 7.0% and showed good stability with maintaining their initial PCE under 1‐sun continuous illumination at maximum power point tracking of 100 h. In addition, quaternary ammonium halide compound (Me_4_NBr) could be used as a passivation layer. In 2019, Du et al, introduced Me_4_NBr to passivate the Sn‐Pb‐based perovskite surface and improved stability.^[^ 80 ^]^

Compared with traditional 3D perovskites, a new so‐called 3D hollow perovskite‐based material is a good candidate for high‐performance solar cells, which created a “hollow” framework by forming a large number of B metal vacancies (and possibly halide X vacancies) in a 3D perovskite “BX_3_” framework.^[^ 81 ^]^ In 2017, Ke et al. reported that ethylenediammonium (en) could serve as an A cation in the 3D FASnI_3_ perovskite structure to form a novel, hollow 3D perovskite {en}FASnI_3_.^[^ 82 ^]^ As a result, en could improve the coverage of the film and inhibited the oxidation of Sn^2+^. This also adjusted the *Eg* of the FASnI_3_ and significantly improved the performance of the {en}FASnI_3_ solar cell, resulting in a maximum PCE of 7.14%. And after aging for over 1000 h with encapsulation, the initial efficiency of 96% was maintained. In the same year, this group explored that en could be universally applied to other perovskites. They used the same method to prepare hollow {en} MASnI_3_ perovskites with an efficiency of up to 6.63%, as shown in Figure 4e.^[^ 83 ^]^ In 2019, they also explored the role of en in tin‐lead mixed perovskites.^[^ 81 ^]^ They found that the {en}FA_0.5_MA_0.5_Sn_0.5_Pb_0.5_I_3_ structure had higher chemical stability than the same structure without en. The results of their study showed that 3D hollow perovskite‐based materials are good candidates for high‐performance, single‐junction solar cells.

In 2019, Yu et al. found that 2,2,2‐Trifluoroethylamine hydrochloride (TFEACl) is an environmentally friendly additive that can be used in conjunction with SnF_2_ to enhance the stability of PSCs.^[^ 84 ^]^ The introduction of Cl^−^ has improved the crystallinity and grain size of the film, and the energy level alignment has been optimized as well. In addition, by incorporating of TFEA^+^ into grain boundaries, the hydrophobicity of the film increases, which can inhibit SnF_2_ segregation. As a result, its PCE increases from 3.63% to 5.3% and its stability is significantly enhanced. Devices with TFEACl still retain more than 60% of the initial PCE (after being left in the air for 350 h), whereas those without TFEACl fails within 120 h under the same test conditions .

#### 4.1.3 Intermediate

The intermediate phase is a Lewis acid‐base adduct formed from a metal halide (used as a Lewis acid) and a polar aprotic solvent (used as a Lewis base).^[^ 85, 86 ^]^ Since tin‐based perovskites tend to crystallize rapidly, uneven and poor quality films are formed. Therefore, the intermediate phase is used to retard crystallization, thereby improving film quality and solar efficiency.

In 2015, Hao et al. used a strong coordinating solvent (DMSO) to form SnI_2_·3DMSO intermediate phase, which could promote uniform nucleation and adjust the growth rate of perovskite film. In this way, a pinhole‐free, uniform MASnI_3_ perovskite film is generated, as shown in Figure 4f.^[^ 40 ^]^ Inspired by this idea, Zhu et al. used trimethylamine (TMA) as an additional Lewis base to explore changes in FASnI_3_ film by two‐step process.^[^ 23 ^]^ They explained that the SnY_2_‐TMA complex not only hinders the rapid reaction between SnI_2_ and FAI but also promotes the uniform dispersion of SnF_2_. As a result, a dense and compact FASnI_3_ film, with large crystal domains, was obtained. Its efficiency increased from 4.34% to 7.09% and showed improved ambient stability. Lee et al. also proposed to enhance the homogeneous dispersion of SnF_2_ by forming the SnF_2_‐pyrazine complex.^[^ 87 ^]^ As introduced with pyrazine, the prepared FASnI_3_ perovskite layer was very smooth and dense without any platy aggregation. This result indicated that pyrazine helps alleviate the phase separation induced by excess SnF_2_ and improves surface morphology of the FASnI_3_ perovskite film.

### 4.2 Deoxidizer

Sn^2+^ is easily oxidized during the preparation process, but adding a suitable reducing agent into the precursor solution can solve this problem well. In addition, reducing agents can also promote the formation of a uniform and dense film. The photovoltaic parameters of different deoxidizer are summarized in **Table** **2**. The effects of several additives are discussed below.

**Table 2:** Photovoltaic parameters of ASnX_3_ PSCs fabricated with different deoxidizer

Deoxidizer	Configuration	*J*_SC_ [mA cm^−2^]	*V*_OC_ [V]	FF [%]	PCE [%]	sPCE [%]	Ref.
HPA	FTO/TiO_2_/Al_2_O_3_/CsSnIBr_2_/Spiro/Au	17.40	0.31	57.00	3.20	—	^[^ 89 ^]^
KHQSA	ITO/NiO*_x_*/FASnI_3_/PCBM/Ag	17.64	0.552	69.40	6.76	5.73	^[^ 92 ^]^
Hydrazine vapor	FTO/TiO_2_/CsSnI_3_/PTAA/Au	30.75	0.17	34.88	1.83		^[^ 93 ^]^
	FTO/TiO_2_/CsSnBr_3_/PTAA/Au	13.96	0.366	59.36	3.04	—	^[^ 93 ^]^
	FTO/TiO_2_/MASnI_3_/PTAA/Au	19.92	0.377	51.73	3.89		^[^ 93 ^]^
N_2_H_5_Cl	ITO/PEDOT:PSS/FASnI_3_/PCBM/BCP/Ag	17.64	0.455	67.00	5.40	4.72	^[^ 94 ^]^
THDH	ITO/PEDOT:PSS/FASnI_3_/PCBM/BCP/Ag	22.12	0.54	50.00	8.48	7.47	^[^ 95 ^]^
Tin powder	ITO/PEDOT:PSS/FASnI_3_/C_60_/BCP/Ag	17.50	0.58	66.30	6.75	5.70	^[^ 97 ^]^

**Figure 5:**
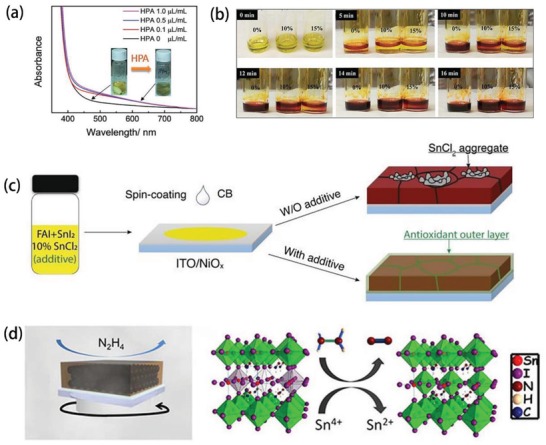
a) UV–visible absorption spectra of CsSnIBr_2_ precursor solution at different HPA concentrations. Reproduced with permission.^[^ 89 ^]^ Copyright 2016, The Royal Society of Chemistry. b) Photograph of FASnI_3_ precursor solution at different mol% AA. Reproduced with permission.^[^ 90 ^]^ Copyright 2017, Science Publishing Group. c) Schematic diagram of a method for preparing perovskite film and the morphology of a perovskite film with or without additives. Reproduced with permission.^[^ 92 ^]^ Copyright 2019, Wiley‐VCH Publications. d) Possible mechanism of reaction of hydrazine vapor with tin‐based perovskite materials. Reproduce with permission.^[^ 93 ^]^ Copyright 2017, American Chemical Society Publications.

Hypophosphorous acid (HPA) is a reducing agent with a P—O bond that coordinates with Sn^2+^.^[^ 88 ^]^ In 2016, Li et al. obtained CsSnIBr_2_ by adding HPA; the best PCE was at 3.2% and showed efficiency‐loss free within 77 d.^[^ 89 ^]^ As a result, the change in color of the precursor solution indicated the formation of a new compound, which was an Sn compound with a cluster structure formed by Sn—O—P—O—Sn coordination bond connection, as shown in **Figure** **5**a. This accelerated the nucleation process and significantly reduced the oxidation of Sn^2+^ during film formation.

Ascorbic acid (AA) is an antioxidant‐acting solution additive, which not only inhibits the oxidation of Sn^2+^ but also modulates binary perovskite crystallization by forming intermediate complexes. In 2017, Xu et al. prepared AA‐treated MA_0.5_FA_0.5_Pb_0.5_Sn_0.5_I_3_ and explored the antioxidant capacity of different AA amounts by storing in the air, as shown in Figure 5b.^[^ 90 ^]^ Its PCE reached 14.01%, which was higher than that when using the SnF_2_ additive one.

The phenolic hydroxyl group can be used as an oxygen scavenger (antioxidant) to protect the interior of the perovskite.^[^ 91 ^]^ Tai et al. prepared FASnI_3_ PSCs by introducing hydroxybenzene sulfonic acid or salt as antioxidant additive and an excess of SnCl_2_.^[^ 92 ^]^ As a result, the SnCl_2_‐additive complex was formed at the grain boundaries, owing to the interaction between SO_3_^−^ and Sn^2+^, as shown in Figure 5c. This composite not only helps eliminate the phase separation caused by SnCl_2_ but also produces in situ encapsulation, which significantly improves the stability of the device. The corresponding PSCs can maintain 80% of efficiency over 500 h upon air exposure without encapsulation.

Hydrazine is a strong reducing agent that may potentially prevent or inhibit the oxidation of Sn^2+^. Adding hydrazine directly to the perovskite solution usually results in complete reduction of the tin halide salt to tin metal. Therefore, a vapor reaction may solve this problem. In 2017, Song et al. presented a feasible method involving a hydrazine vapor atmosphere with a conventional SnF_2_ additive during the preparation of tin‐based halide PSCs.^[^ 93 ^]^ As a result, the possible reduction processed path was: 2SnI_6_^2−^ + N_2_H_4_ → 2SnI_4_^2−^ + N_2_ + 4HI, as shown in Figure 5d. In this way, the oxidation of Sn^2+^ was reduced and the PCE of FASnI_3_, MASnI_3_, and CsSnI_3_ PSCs was significantly improved, indicating that this is a versatile method. In the same year, they used the same method to prepare CsSnI_3_ with excess SnI_2_, achieving an efficiency of 4.81%.^[^ 72 ^]^ In addition to using the above hydrazine vapor, a small amount of hydrazinium chloride (N_2_H_5_Cl) and SnF_2_ codoped can prevent the direct reduction of tin. Kayesh et al. obtained a uniform and pinhole‐free FASnI_3_ film by adding 2.5 mol% N_2_H_5_Cl in this method.^[^ 94 ^]^ As a result, the content of Sn^4+^ was reduced by 20%, and the best PCE obtained was 5.4%, which retains 65% of its original PCE for up to 1000 h without encapsulation. In addition to inhibiting the oxidation of Sn^2+^, such reducing agents can also optimize morphology. For example, Li et al. used trihydrazine dihydriodide (THDH) as an additive for solution deposition of FASnI_3_ perovskite layer.^[^ 95 ^]^ THDH is effective in reducing the amount of Sn^4+^ caused by the release of hydrazine from THDH in solution. Moreover, the hydrazinium iodide (N_2_H_5_I) left by THDH promoted uniform film formation and obtained dense FASnI_3_ film. As a result, a maximum PCE of 8.48% was achieved in a planar heterojunction PSC.

Tin powder can be used as a reducing agent. Lin et al. used metallic tin to reduce the oxidation of Sn^2+^ to Sn^4+^ and prepared a narrow bandgap Pb‐Sn perovskite, yielding a 24.8% PCE.^[^ 96 ^]^ Adding tin to the precursor solution can cause Sn^4+^ + Sn → 2Sn^2+^. Gu et al. also put the Sn powder into the FASnI_3_ precursor solution.^[^ 97 ^]^ As a result, the best PCE was at 6.75%, which was higher than the FASnI_3_‐based PSCs with SnF_2_ as the only additive or device fabricated from SnI_2_ of 99.999% purity. These findings demonstrated the importance of pure SnI_2_ and showed that it was a good way to purify the tin source with tin powder.

### 4.3 Partial Substitution

Ion doping is often optimized for device performance.^[^ 98 ^]^ The photovoltaic parameters of partial substituted ASnX_3_‐based PSCs are summarized in **Table** **3**. In improving the stability of tin‐based PSCs, its role is mainly as follows:

1) Adjust the tolerance factor to stabilize the crystal structure
2) Optimize the morphology of the film and form a passivation film to enhance the stability of the perovskite

**Table 3:** Photovoltaic parameters of partial substituted ASnX_3_ based PSCs

Configuration	*J*_SC_ [mA cm^−2^]	*V*_OC_ [V]	FF [%]	PCE [%]	sPCE [%]	Ref.
ITO/PEDOT:PSS/Cs*_X_*FA_1−_ *_X_*SnI_3_/C_60_/BCP/Ag	20.70	0.44	66.80	6.08	—	^[^ 100 ^]^
ITO/PEDOT:PSS/MA_(1−_ *_X_*_)_HA*_X_*SnI_3_/BCP/Ag	14.10	0.38	49.00	2.60	2.10	^[^ 101 ^]^
FTO/TiO_2_/MASnI_3_/PTAA/Au	22.91	0.486	64.00	7.13	6.32	^[^ 102 ^]^
ITO/PEDOT:PSS/FA_0.75_MA_0.25_Sn_1−_ *_X_*Ge*_X_*I_3_/C_60_/BCP/Ag	19.50	0.42	41.00	4.48	—	^[^ 105 ^]^
ITO/PEDOT:PSS/FA_0.75_MA_0.25_Sn_1−_ *_X_*Ge*_X_*I_3_/C_60_/BCP/Ag/Au	25.3	0.44	71	7.90	—	^[^ 106 ^]^
FTO/PCBM/CsSn_0.5_Ge_0.5_I_3_/Spiro/Au	18.41	0.63	61.3	7.11	7.03	^[^ 107 ^]^
FTO/TiO_2_/FASn(Br*_X_*I_1−_ *_X_*)_3_/Spiro/Au	19.80	0.414	66.90	5.50	5.00	^[^ 109 ^]^

**Figure 6:**
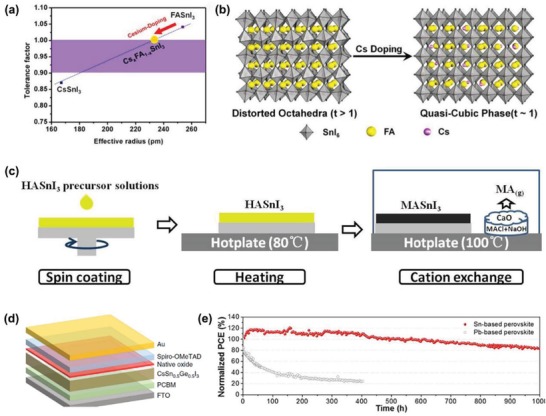
a) Correlation between the tolerance factor of the Cs*_x_*FA_1−_ *_x_*SnI_3_ perovskite and the effective radius of the Cs/FA cation. b) Schematic diagram of the structural transformation of FASnI_3_ when appropriate Cs is incorporated. Reproduce with permission.^[^ 100 ^]^ Copyright 2018, American Chemical Society Publications. c) Schematic diagram of the cation exchange process. Reproduced with permission.^[^ 102 ^]^ Copyright 2019, Wiley‐VCH Publications. d) Schematic diagram of planar PSC structure. Reproduced with permission.^[^ 107 ^]^ Copyright 2019, Nature Publishing Group. e) Normalized PCE of the encapsulated tin‐based and Pb‐based PSCs was under continuous illumination for 1000 h. Reproduce with permission.^[^ 109 ^]^ Copyright 2016, American Chemical Society Publications.

FASnI_3_'s tolerance factor was 1.04, which was larger than 1, indicating that it deviates from the rational perovskite structure and leads to material instability.^[^ 99 ^]^ In 2018, Gao et al. introduced Cs^+^ into the FASnI_3_ lattice to shrink the lattice with a tolerance factor *t* close to 1, as shown in **Figure** **6**a,b.^[^ 100 ^]^ As a result, the optimum PCE was 6.08%, which was 63% higher than that of the control device (3.74%). Oxidation of Sn^2+^ was inhibited and good stability was obtained. After 2000 h of storage in N_2_ atmosphere, the initial PCE remained at 90%. The method was aimed to structurally stabilize the perovskite by adjusting tolerance coefficient.

The following method optimizes the film's morphology by ions exchange and releases a reducing gas, which can improve stability and optimize performance. Hydrazinium (HA^+^) is similar to MA^+^ and has a reducing property to prevent oxidation and disproportionation of Sn^2+^. In 2018, Tsarev et al. prepared MA_(1−_ *_x_*_)_HA*_x_*SnI_3_. The addition of HA improved the morphology of the film and performance of the device.^[^ 101 ^]^ However, the best efficiency of the device was at only 2.6%. In the next year, Li et al. prepared a dense and uniform MASnI_3_ film by a two‐step process, as shown in Figure 6c.^[^ 102 ^]^ The reaction by in situ organic cation exchange reaction is as follows: CH_3_NH_2_ (g) + NH_2_NH_3_SnI_3_ (s) → CH_3_NH_3_SnI_3_ (s) + NH_2_NH_2_ (g). Released HA gas can inhibit the oxidation of Sn^2+^. Its efficiency has been greatly improved, with the best efficiency obtained at 7.13%.

Partial ion substitution at position B can also optimize tin‐based properties. Germanium (Ge) and tin (Sn) are the same group of elements, located above Sn, Ge can replace Sn, thereby improving the performance and stability of Tin‐based perovskite. Germanium PSCs were first reported in 2015, with a PCE of 0.22% methylammonium germanium iodide perovskite (MAGeI_3_).^[^ 103 ^]^ Inspired by this study, in 2018, Trimmel et al. introduced bromide ions into MAGeI_3_ to obtain a PCE of 0.57% (MAGeI_2.7_Br_0.3_), while slightly improving the stability of germanium perovskite.^[^ 104 ^]^ In the same year, Ito et al. added 5% Ge into the FA_0.75_MA_0.25_SnI_3_ perovskite; its PCE increased from 3.31% (pure tin‐based perovskite) to 4.48% (6.90% after 72 h) when measured in air without encapsulation.^[^ 105 ^]^ In the next year, Ng et al. explored the effect of germanium (Ge) on passivation and reducing trap states.^[^ 106 ^]^ They found that in the optimal Ge state, the trap density decreased from 10^15^–10^17^ cm^−3^ (without Ge) to 10^8^–10^14^ cm^−3^ and exhibited a longer carrier lifetime. The optimal PCE of the device was 7.9%. Recently, Chen et al. proposed a new natural oxide passivation method to improve the efficiency and stability of lead‐free PSC.^[^ 107 ^]^ They prepared CsSn_0.5_Ge_0.5_I_3_ perovskite film. The extremely high oxidation activity of Ge(II) enabled the rapid formation of an ultrathin uniform native‐oxide surface passivating layer, as shown in Figure 6d. This natural oxide was GeO_2_ doped with a small amount of Sn, which could inhibit the recombination of photo carriers at the interface and enhance the inherent or thermodynamic stability of perovskite by passivating properties of the natural oxide. As a result, they obtained the optimum PCE of 7.11% and high stability, with less than 10% decay in efficiency after 500 h of continuous operation in N_2_ atmosphere under 1‐sun illumination.

The mixed halide perovskite can not only change the crystal structure and adjust the bandgap,^[^ 43 ^]^ but also improve its humidity stability.^[^ 108 ^]^ Lee et al. introduced bromide (Br) ions into the FASnI_3_ lattice, which significantly reduces the carrier density.^[^ 109 ^]^ This is considered to be the case where Br is present, in which the formation of Sn vacancy defects can be suppressed effectively. FASnI_3_ PSC doped with Br (25 mol%) exhibits a device efficiency of 5.5% and shows good light stability, maintaining an initial efficiency of 83% under 1000 h of illumination as shown in Figure 6e.

Recently, Pisanu et al. explored polycrystalline powder samples of the new MA_1−_ *_x_*DMA*_x_*SnBr_3_ system in which DMA is dimethylammonium.^[^ 110 ^]^ They explored its stability by placing the sample in humid air. The results showed that DMASnBr_3_ had strong resistance to the oxidation of Sn(II). This is a new method for inhibiting the oxidation of Sn^2+^ and was expected to be applied to tin‐based PSCs.

### 4.4 Low‐Dimensional Tin‐Based Perovskites

LD perovskites are more stable than 3D perovskites; as such, the former are a good way to improve the stability of tin‐based perovskites.^[^ 111, 112, 113 ^]^ LD perovskites include 2D, mixed‐phase (2D/3D), and quantum dots (QD). We will introduce them in detail based on these three aspects. **Table** **4** presents the photovoltaic parameters of LD tin‐based perovskites.

**Table 4:** Photovoltaic parameters of low dimensional based ASnX_3_ PSCs

Materials dimensional	Configuration	*J*_SC_ [mA cm^−2^]	*V*_OC_ [V]	FF [%]	PCE [%]	sPCE [%]	Ref.
2D	FTO/TiO_2_/(BA)_2_(MA)*_n_*_−1_Sn*_n_*I_3_ *_n_*/PTAA/Au	24.10	0.229	45.70	2.50	2.43	^[^ 14 ^]^
	ITO/PEDOT:PSS/BA_2_MA_3_Sn_4_I_13_/PCBM/Al	21.87	0.38	48.30	4.03		^[^ 117 ^]^
	ITO/NiO*_x_*/(PEA)_2_(FA)*_n_*_−1_Sn*_n_*I_3_ *_n_*_+1_/PCBM/Al	14.44	0.59	69.00	5.94		^[^ 118 ^]^
	ITO/PEDOT:PSS/(PEA)_2_(FA)*_n_*_−1_Sn*_n_*I_3_ *_n_*_+1_/PC_60_BM/Al	21.80	0.53	66.50	8.17	7.66	^[^ 119 ^]^
	ITO/PEDOT:PSS/(BA_0.5_PEA_0.5_)_2_FA_3_Sn_4_I_13_/C_60_/Al	21.82	0.60	66.73	8.82		^[^ 120 ^]^
	ITO/PEDOT:PSS/AVA_2_FA*_n_*_−1_Sn*_n_*I_3_ *_n_*_+1_/PCBM/BCP/Ag	21.00	0.61	68.00	8.71		^[^ 122 ^]^
	ITO/TiO_2_/Al_2_O_3_/HEA*_x_*FA_1−_ *_x_*SnI_3_/Carbon	18.52	0.371	56.20	3.90		^[^ 123 ^]^
2D/3D	ITO/PEDOT:PSS/2D/3D‐based FASnI_3_/C_60_/BCP/Cu	24.87	0.45	63.00	7.05	6.30	^[^ 124 ^]^
	ITO/PEDOT:PSS/2D/3D‐based FASnI_3_/C_60_/BCP/Al	24.10	0.525	71.00	9.00		^[^ 125 ^]^
	ITO/NiO*_x_*/2D‐quasi‐2D–3D PEA_0.15_FA_0.85_SnI_3_/PCBM/BCP/Ag	22.00	0.61	70.10	9.41	8.75	^[^ 126 ^]^
	ITO/PEDOT:PSS/EA*_X_*PEA_2_FASn_2_I_7_/C_60_/BCP/Al	23.75	0.51	70.00	8.40	7.87	^[^ 127 ^]^
QDs	ITO/PEDOT:PSS/CsSnI_3_/PCBM/Ag	23.79	0.42	41.3	4.13	4.13	^[^ 132 ^]^

#### 4.4.1 2D

In lead‐based perovskites, 2D films have been shown to be more moisture‐resistant than 3D films.^[^ 114 ^]^ The layered structure in a 2D perovskite inhibits ion migration and enhances moisture resistance by introducing a long cationic chain with hydrophobicity, inhibiting moisture, and oxygen from entering the film, thereby improving stability.^[^ 115 ^]^ In addition, the crystallization of a 2D perovskite can inhibit the formation of defects, which, in turn, contributes to low self‐doping levels.^[^ 116 ^]^ Based on LD perovskites' advantages mentioned above, many researchers have begun to explore 2D tin‐based PSCs to improve their stability.

The organic cations of n‐butylamine (BA) and phenethylamine (PEA) are often used in 2D perovskites. In 2017, Gao et al. prepared a 2D Ruddlesden–Popper (RP) (BA)_2_(MA)*_n_*_−1_Sn*_n_*I_3_ *_n_* perovskite film.^[^ 14 ^]^ They found that different solvents could change the orientation of the film, such as parallel or vertical. In addition, they introduced triethylphosphine as an effective antioxidant and intermediate ligand to improve the morphology of the film and inhibited the oxidation of Sn^2+^. As a result, the best PCE was 2.5% (*n* = 4). The encapsulated device maintained an initial 90% after 1 month and dropped to 50% after 4 months; stability was greatly improved. Later, Qiu et al. reported for the first time the crystallization kinetics management of RP tin‐based perovskites controlled by Lewis adducts and ion exchange processes.^[^ 117 ^]^ As a result, they obtained a film with a good average grain size of ≈9 µm; the best PCE was at 4.03%. It showed good stability and was placed in a nitrogen atmosphere for 94 d without degradation.

**Figure 7:**
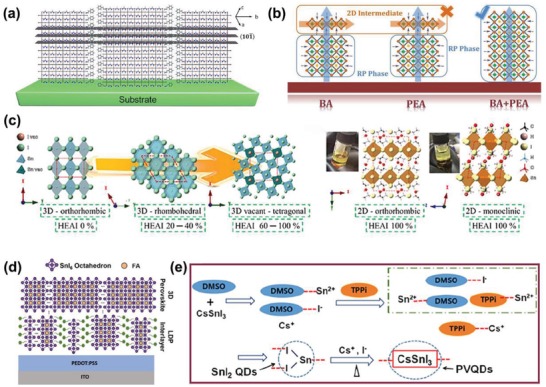
a) Schematic illustration of the (101) plane of a (PEA)_2_(FA)_8_Sn_9_I_28_ (*n* = 9) 2D perovskite crystal. Reproduce with permission.^[^ 118 ^]^ Copyright 2017, American Chemical Society Publications. b) Schematic diagram of 2DRP Sn perovskite crystal growth based on BA, PEA, and BA+PEA. Reproduce with permission.^[^ 120 ^]^ Copyright 2019, American Chemical Society Publications. c) The perovskite structures of single crystals with HEAI proportions 0%, 40%, 80%, and 100% represented from left to right. Reproduce with permission.^[^ 123 ^]^ Copyright 2018, American Chemical Society Publications. d) Schematic illustration of the LDP at the interface. Reproduced with permission.^[^ 124 ^]^ Copyright 2018, Science Publishing Group. e) Scheme of the production process of the PVQDs. Reproduced with permission.^[^ 132 ^]^ Copyright 2019, The Royal Society of Chemistry.

In 2017, Liao et al. prepared (PEA)_2_(FA)*_n_*_−1_SnI_3_ *_n_*_+1_ perovskite films; by changing the ratio of PEA, they achieved a highly directional growth of the film perpendicular to the substrate, as shown in **Figure** **7**a.^[^ 118 ^]^ The corresponding PCE for a PSC was at 5.94% and had been maintained for 1000 h without encapsulation. Later, Kim et al. explored the effect of formamidinium thiocyanate additive on quasi‐2D perovskites.^[^ 119 ^]^ They found that SCN^−^ could react strongly to Sn^2+^, to inhibit the formation of Sn^4+^, and improve the quality of the film, to obtain a dense, uniform, pinhole‐free film. As a result, the maximum efficiency under reverse scanning was at 8.17%, which was reduced by only 10% after 1000 h of storage in the glove box. Later, Qiu et al. explored the synergy between BA and PEA to control the crystallization process.^[^ 120 ^]^ The interaction of BA and PEA could effectively inhibit the formation of mesophase during crystal growth, as shown in Figure 7b. As a result, a high‐quality thin film was obtained and the crystal orientation was improved. The efficiency also improved to 8.82%, with good stability.

5‐ammonium valerate organic spacer cations (5‐AVA^+^) can be used in lead‐based 2D PSCs to provide high stability and high performance.^[^ 121 ^]^ Inspired by this idea, Xu et al. prepared AVA_2_FA*_n_*_−1_Sn*_n_*I_3_ *_n_*_+1_ (<*n*> = 5) films by NH_4_Cl additives.^[^ 122 ^]^ As a result, the orientation and morphology of a tin‐based perovskite film could be achieved by adjusting the amount of NH_4_Cl additive. When the amount of NH_4_Cl was 10%, a highly vertical‐oriented film could be obtained and efficiency increased from 4.19% to 8.71%, with an excellent stability, which can maintain its initial performance for over 400 h without significant attenuation.

Tsai et al. also explored the control of crystal structure by HEA^+^ cations (2‐hydroxyethylammonium).^[^ 123 ^]^ As the proportion of HEAI increases, the crystal structure changed from 3D to 2D, as shown in Figure 7c. The best PCE was at 3.7% when the HEAI was 40% (*x* = 0.4). Efficiency increased to 3.9% after 340 h of storage in the glove box.

#### 4.4.2 2D/3D

The 2D/3D perovskites combine a highly stable 2D perovskite with a 3D perovskite of a full color absorption and excellent charge transport to achieve an efficient and stable PSCs.^[^ 79 ^]^ In 2018, Chen et al. introduced an LD perovskite interlayer near the p‐type interface in an inverted FASnI_3_ PSC, which reduced the trap state and suppressed charge recombination.^[^ 124 ^]^ At the interface, an ultrathin interlayer, consisting of a 2D structure or a 2D–3D mixture, will be formed. The upper perovskite away from the interface will retain the original 3D structure, as shown in Figure 7d. In addition, the LD perovskite interlayer has a trap passivation effect to help suppress the accumulation and recombination of carriers, which, in turn, leads to efficient extraction of carriers. As a result, the maximum PCE achieved is at 7.05%, with stabilized power output and negligible *J–V* hysteresis. Shao et al. also prepared 2D/3D‐based FASnI_3_ perovskite and obtained a highly crystalline film, with a maximum efficiency of 9.2%.^[^ 125 ^]^ Compared with the best PCE for pure FASnI_3_ film, its PCE had a 50% increase when SnF_2_ is used as a reducing agent.This is because it has very low trap assisted recombination, low shunt losses, and more efficient charge collection. Different from the above structure, Wang et al. introduced removable pseudohalogen ammonium thiocyanate (NH_4_SCN) to manipulate the crystal growth. A parallel‐oriented 2D perovskite on the surface of the film was formed, which significantly improved the stability and oxidation resistance of FASnI_3_ perovskite with its efficiency increased to 9.41%.^[^ 126 ^]^ The device can retain 90% of its initial performance for nearly 600 h. Pinholes may be generated in 2D/3D perovskite films, which may limit the performance of the device. Shao et al. obtained a more uniform EA*_x_*2D/3D film by using ethylammonium iodide (EAI) as an additive, which successfully reduced the number of defects in the film, and improved the performance of the device to 8.4%.^[^ 127 ^]^

#### 4.4.3 QDs

Quantum dot methods are important in reducing intrinsic defects caused by large surface‐to‐volume ratios and automatic elimination of volume defects.^[^ 128, 129, 130 ^]^ To improve the stability of tin‐based perovskites, Liu et al. synthesized the CsSn_1−_ *_x_*Pb*_x_*I_3_ perovskite quantum dot (PVQDs) using a simple method that can keep intact when directly exposed to the ambient air.^[^ 131 ^]^ Later, Wang et al. synthesized pure CsSnI_3_ PVQD using triphenyl phosphite (TPPi) as an antioxidant as shown in Figure 7e.^[^ 132 ^]^ As a result, a maximum efficiency of 5.03% was achieved in a solar cell based on CsSnI_3_ PVQD, which opened up new avenues for high‐performance preparation of tin‐based perovskites.

## 5 Conclusion and Prospect

In summary, the nontoxic tin‐based perovskite has great development prospects in the future. However, its lack of instability prevents its further development. Although great progress has been made, difficulties, such as inefficiency and instability, limit its commercialized development. Therefore, based on our analysis, we will provide suggestions on ways to promote the stability of tin‐based PSCs

- Improving pure tin halide precursor materials. Preparation and use of high‐purity tin halide precursor materials can prevent oxidation during film processing, especially purify the tin source.^[^ 133 ^]^
- Decreasing nonradiative recombination rate and optimizing the *V*_OC_ of tin‐based PSCs. A major obstacle limiting the development of tin‐based perovskites is the loss of *V*_OC_. As can be seen in Tables 1, 2, 3, 4, the *V*_OC_ value is generally lower than 0.6 eV, which is mainly due to the existence of severe recombination and mismatched energy levels in the device. Therefore, controlling the crystallization process of perovskite to reduce trap state is another strategy for developing tin‐based perovskites in the future. For example, Meng et al. achieved a high *V*_OC_ (0.63 V) by introducing poly(vinyl alcohol) to FASnI_3_.^[^ 134 ^]^
- Reducing exposure to oxygen. Since Sn^2+^ is more likely to begin to oxidize from the surface and grain boundaries of the perovskite film, forming an antioxidant protective layer on perovskite particles or surface may also be an effective way to improve the efficiency and stability of tin‐based PSCs.^[^ 30, 77 ^]^ This strategy should be researched further and developed in the future. Timely encapsulation can avoid oxygen, thereby improving stability as well.
- Reducing the perovskite dimension. Combining with the stability and low defects of LD perovskites, the inherent oxidation problems of tin‐based perovskites can be improved.^[^ 125 ^]^ However, LD tin‐based perovskites on different additives and reducing agents have commanded less research, allowing for more explorations on their applications.^[^ 119 ^]^ Meanwhile, the development of new LD materials, such as (2‐(4‐(3‐fluoro)stilbenyl) ethanammonium iodide (FSAI)),^[^ 135 ^]^ is another strategy to further improve their performance in the future. Finally, there are a few studies on Dion–Jacobson and interlayer space in tin‐based PSCs, pointing us to a new development path.
- Exploring compact inorganic transport layer. Organic matter is not good at isolating water and oxygen and easy to deteriorate, which is detrimental to the easy oxidation of tin group. Therefore, the development of an all inorganic PSC, such as an inorganic electron transport layer and hole transport layer,^[^ 136, 137 ^]^ can further isolate oxygen and moisture and inhibit the oxidation of Sn^2+^.
- Developing inverted tin‐based PSCs. The reported device mainly based on the traditional structure. Recently, Diau et al. summarized the properties of inverted and normal tin‐based PSCs, and found the inverted structure has high and stable performance, and Sn^2+^/Sn^4+^ oxidation is even less obvious in inverted devices.^[^ 138 ^]^ So, constructing the inverted structure and combining the current new hole transport layer^[^ 139 ^]^ and electron transport layer^[^ 140 ^]^ to develop an efficient and stable tin‐based PSCs should be an efficient approach.

In conclusion, nontoxic PSCs are a major trend in the future, and the development prospect is very optimistic. The lead‐based PSCs have developed rapidly, from the initial efficiency of 3.8%^[^ 1 ^]^ to the current efficiency of 25.2%^[^ 2 ^]^ within a span of only 10 years. The tin‐based PSCs have developed well in only few years. Therefore, we should treat tin‐based PSCs with an optimistic attitude and believes that this field will develop well in the future.

## Conflict of Interest

The authors declare no conflict of interest.
